# Regulatory T Cell Induced by* Poria cocos* Bark Exert Therapeutic Effects in Murine Models of Atopic Dermatitis and Food Allergy

**DOI:** 10.1155/2016/3472608

**Published:** 2016-06-29

**Authors:** Min-Jung Bae, Hye-Jeong See, Gyeyoung Choi, Chang-Yuil Kang, Dong-Hwa Shon, Hee Soon Shin

**Affiliations:** ^1^Institutes of Entrepreneurial BioConvergence, Seoul National University, Seoul 151-742, Republic of Korea; ^2^Division of Nutrition and Metabolism Research, Korea Food Research Institute, Bundang-gu, Seongnam-si, Gyeonggi-do 463-746, Republic of Korea; ^3^WCU Department of Molecular Medicine and Biopharmaceutical Science, Graduate School of Convergence Science and Technology and College of Pharmacy, Seoul National University, Seoul 151-742, Republic of Korea; ^4^Laboratory of Immunology, Research Institute of Pharmaceutical Sciences, College of Pharmacy, Seoul National University, Seoul 151-742, Republic of Korea; ^5^Food Biotechnology Program, Korea University of Science and Technology, Daejeon 305-350, Republic of Korea

## Abstract

The prevalence of allergic disorders including atopic dermatitis (AD) and food allergy (FA) has increased dramatically in pediatric populations, but there is no effective drug available for their management. Therefore, trials are required for the development of safe therapeutic agents such as herbal medicines. We determined whether orally administered* Poria cocos* bark (PCB) extract could exert immunosuppressive effects on allergic and inflammatory symptoms of AD and FA. For both AD, which was induced using house dust mite extract, and FA, which was induced by exposure to ovalbumin, model mice were orally treated with PCB extract for 62 days and 18 days, respectively. We also investigated the inductive effect of PCB extract on the generation and maintenance of Foxp3^+^CD4^+^ regulatory T cells (Tregs). The symptoms of AD and FA were ameliorated by the administration of PCB extract. Furthermore, PCB extract inhibited the Th2-related cytokines and increased the population of Foxp3^+^CD4^+^ Tregs in both AD and FA models. In* ex vivo* experiments, PCB extract promoted the functional differentiation of Foxp3^+^CD4^+^ Tregs, which is dependent on aryl hydrocarbon receptor activation. Thus, PCB extract has potential as an oral immune suppressor for the treatment of AD and FA through the generation of Tregs.

## 1. Introduction

Hypersensitivity diseases called allergic disorders result from the breakdown of natural tolerance, and their prevalence has increased worldwide. In particular, the incidence of atopic dermatitis (AD) and food allergy (FA) is increasing and they have been estimated to affect 10–20% and 5-6% of the pediatric population, respectively [1, 2].

AD is a chronic and allergic inflammatory skin disease [3] caused by both genetic and environmental factors [2]. In general, AD develops owing to sensitization of allergens such as food and house dust mite (HDM), and then abnormal patterns in the immune system contribute to the pathogenesis and development of AD [4]. In particular, T-helper type 2- (Th2-) dominant immune responses play key roles in AD. The number of various immune cells such as T lymphocytes, macrophages, eosinophils, and mast cells increases in AD and these cells infiltrate to form skin lesions. Therefore, these cells could be targeted to treat or prevent AD [5, 6].

FA is a hypersensitivity disorder caused by substances derived from foods and develops when the immune system reacts to food allergens after ingestion. Food allergens such as ovalbumin (OVA) and ovomucoid from eggs, caseins and beta-lactoglobulin from milk, peanut agglutinin from peanuts, and tropomyosins from shrimp are known [7]. Mild allergic reactions are common, causing symptoms such as diarrhea, hypotension, pruritus, and vomiting [8]. However, in severe cases, lethal anaphylactic shock can occur [9].

Regulatory T (Treg) cells and Forkhead box P3- (Foxp3-) expressing CD4^+^ T cells are important for the functions of the immune system. Since functional Foxp3^+^CD4^+^ T cells have immune suppressive activities, they are able to attenuate hyperimmune responses and the inflammatory, allergic, and autoimmune disorders due to these responses [10–12]. Therefore, the induction of* Foxp3* expression in these diseases may contribute to the induction of immune tolerance by generation of regulatory T cells [13] and suppress the symptoms of HDM-induced AD and OVA-induced FA.

The generation of Foxp3^+^ Tregs is driven by transforming growth factor-*β* (TGF-*β*) and interleukin-2 (IL-2) [14, 15]. Recent studies concluded that Treg generation is promoted by ligand-dependent activity and the expression of aryl hydrocarbon receptor (AhR), which is recruited to the* Foxp3* promoter [16]. Natural AhR ligands can be isolated or synthesized from dietary compounds including curcumin, indirubin, or indole-3-carbinol (I3C), as well as 2,3,7,8-tetrachlorodibenzo-*p*-dioxin [17–19]. The finding that natural ligands affect the induction of Foxp3^+^ Tregs via AhR activation is receiving increased attention [20, 21]. Therefore, these dietary AhR ligands might influence the onset of AD and FA.

*Poria cocos* is an edible basidiomycete that grows on pine tree roots; it is widely used as a traditional medicine in East Asia. The* P. cocos* extract has beneficial effects on inflammation and cancer [22]. Recent studies have focused on the beneficial effects of* P. cocos* derivatives on immune function [23–25]. In particular, the* P. cocos* bark (PCB) extracts include triterpenoid compounds or/and ergosterol, which is used to treat inflammatory diseases including rheumatoid arthritis and systemic lupus erythematosus and symptoms associated with ulcerative colitis [26, 27]. However, the scientific evidence for the antiallergic effects of PCB extract is not clear.

The aim of this study was to determine whether PCB extract exerts an effect on the generation and maintenance of Foxp3^+^CD4^+^ Tregs. Furthermore, we investigated whether orally administered PCB extracts could improve allergic symptoms in murine models of HDM-induced AD and OVA-induced FA.

## 2. Materials and Methods

### 2.1. Sample Preparation

The PCB extract used in this study was from the Plant Extract Bank of Korea (Plant Diversity Research Center of the 21st Century Frontier Research Program). Voucher specimens were deposited at the herbarium of the Plant Extract Bank of Korea (Daejeon, Korea). Ethyl alcohol (EtOH; 95%) dried extracts were dissolved in saline (Sigma-Aldrich, St. Louis, MO) and dimethylsulfoxide (DMSO) before use. Endotoxin level in PCB extract using Pyrogent Plus assay kit (Lonza, Hopkinton, Massachusetts) was found to be below 0.06 EU/mL, which is the limit of sensitivity for this kit.

### 2.2. Animal Experiments

Four-week-old female BALB/c mice (18–20 g) were purchased from OrientBio Inc. (Kyeonggi, Korea) and housed at 23 ± 2°C with a 12 h light/dark cycle and free access to food and water. This study was carried out in strict accordance with the recommendations in the Guide for the Care and Use of Laboratory Animals of the National Institutes of Health with special attention to minimizing animal pain. The study protocol was approved by the Animal Care and Use Committee of Korea Food Research Institute (Approval Number: KFRI-M-14018). No animals were sacrificed or died as a result of the experiment before day 35. Six-week-old female Foxp3-GFP C57BL/6 mice were previously described [28]. These mice were housed at 23 ± 2°C with a 12 h light/dark cycle and free access to food and water. All experimental procedures were performed in compliance with the guidelines set by the University Animal Care and Use Committee at Seoul National University with special attention to minimizing animal pain. The study protocol was approved by the Seoul National University Institutional Animal Care and Use Committee (Approval Number: SNU-130807-4-1).

### 2.3. Cell Purification and Sorting

Naïve CD4^+^ T cells from BALB/c mice were isolated using a magnetic-activated cell sorting (MACS) CD4^+^CD62L^+^ T cell isolation kit (Miltenyi Biotec, Bergisch-Gladbach, Germany) following the manufacturer's instructions. T cell-depleted cells, as antigen-presenting cells (APCs), were prepared from the spleen cells of BALB/c mice and mLN cells by negative selection using MACS. The purity of isolated cells was confirmed using FACS. Foxp3^+^CD4^+^ T cells were sorted from Foxp3eGFP to >99% purity using FACS Aria III (BD Biosciences) with anti-CD4, anti-CD45RB, and anti-CD25 antibodies (eBioscience). On day 3, the Treg population was isolated based on CD4 and Foxp3-eGFP expression using FACS.

### 2.4. Functional Differentiation of Naïve CD4^+^ T Cells

Naïve BALB/c CD4^+^ T cells (1 × 10^6^ cells/well) were cultured in 96-well plates in the presence of 0–100 mg/mL PCB extract in 0.1% DMSO. T cell receptor (TCR) stimulation was performed using 10 *μ*g/mL plate-bound anti-CD3 (BioLegend, San Diego, CA, 17A2) and 2 *μ*g/mL soluble anti-CD28 (BioLegend, San Diego, CA, 37.51) antibodies. To analyze the CFSE^+^ population that was converted by Foxp3^+^CD4^+^ Treg, CD4^+^CD62L^+^ T cells labeled with 5 *μ*M CFSE (Life Technologies, Australia) were cocultured with CD4^+^ cells that had been precultured in the presence or absence of PCB extract and/or TCR stimulation for 3 days. The CFSE^+^ population was then analyzed using FACS.

### 2.5. Induction of AD by Administration of HDM Extract in the Ear

**Figure 1:**
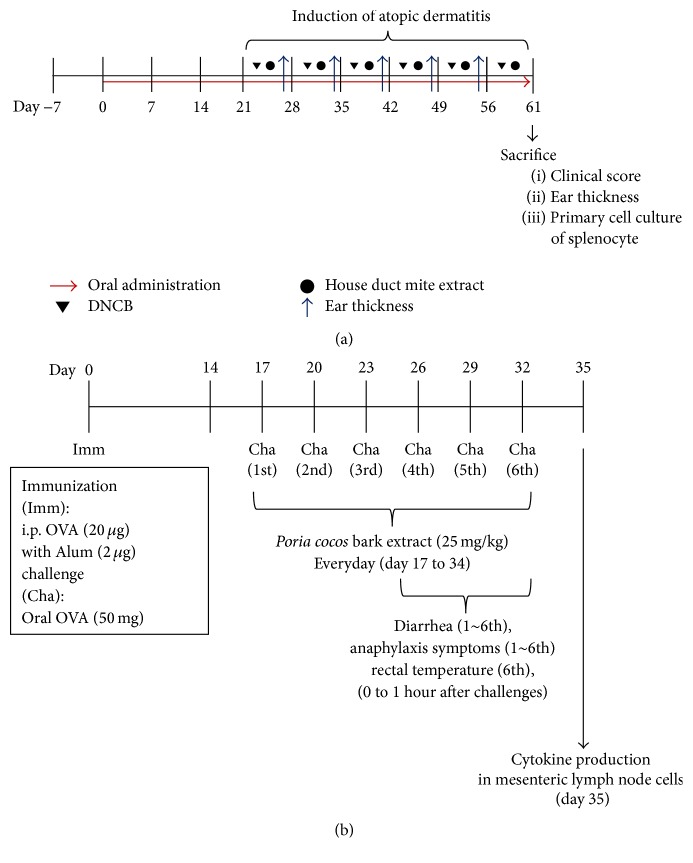
Experimental designs for inductions of atopic dermatitis and food allergy. (a) Five-week-old female BALB/c mice were divided into naïve (*n* = 7), sham (*n* = 12), PCB (*n* = 12), and dexamethasone (*n* = 8) groups. 25 mg/kg of PCB extract was orally administrated from day 0 to 61. For induction of atopic dermatitis, each 1% DNCB and 10 mg/mL of HDM extract was applied to each ear once a week from day 21 (total six times). (b) Six-week-old female BALB/c mice were divided into naïve (*n* = 5), sham (*n* = 9), PCB (*n* = 9), and dexamethasone (*n* = 5) groups. To induce food allergies, mice were immunized with 20 *μ*g OVA and 2 mg Imject Alum by intraperitoneal (i.p.) injection on day 0. From day 17, mice were orally challenged with 50 mg OVA in saline every 3 days, for a total of six times. Next, 25 mg/kg PCB extract was administered orally daily from day 17 to 34.

The mice were divided into naïve (*n* = 8), sham (*n* = 11), PCB (*n* = 8), and dexamethasone (*n* = 6) groups. To induce AD, the surfaces of both ear lobes of the mice were stripped using surgical tape (Shinshin Pharm., Korea). After stripping, 20 *μ*L of 1% 2,4-dinitrochlorobenzene (DNCB) (Sigma-Aldrich, St. Louis, MO, USA) dissolved in acetone/olive oil solution (acetone : olive oil  =  1 : 3) was painted on each ear lobe. After 2 days, 20 *μ*L of HDM extract (10 mg/mL,* Dermatophagoides farinae*, GREER Source Materials, Lenoir, NC, USA) dissolved in phosphate-buffered saline (PBS) was reapplied on each ear lobe. Challenges with DNCB and HDM extract were repeated once a week alternatively until 6 weeks; the naïve group was challenged with only tape stripping and PBS. PCB extract (25 mg/kg) was administered orally daily for 3 weeks before AD was induced by HDM and DNCB (Figure 1(a)). Clinical score was calculated using modified criteria [29]. Briefly, each ear region was assessed separately for erythema/edema, scaling/dryness, and excoriation/hemorrhage, and the average degree of severity in each ear region was assigned a score of 0, 1, 2, or 3, which indicated none, mild, moderate, and severe, respectively.

### 2.6. Analysis of Histological Changes in the Ear

Ear lesions of each group were fixed with 10% formalin solution and embedded in paraffin. They were then cut into 6 *μ*m thick sections and stained with hematoxylin (Sigma-Aldrich, St. Louis, MO, USA) and eosin (Sigma-Aldrich, St. Louis, MO, USA) solution (H&E). H&E staining was performed according to standard procedures (IHC World, Online Information Center for Histochemistry; http://www.ihcworld.com/). All histologic analyses were performed by a professional pathologist at the College of Veterinary Medicine, Seoul National University. Analysis of stained sections was performed using a microscope at 100x and 200x magnification.

### 2.7. Isolation and Analysis of Infiltrated Cells in the Ears

Ear tissues were removed and placed in 1.0 mmol/L EDTA in RPMI 1640 medium. The ear tissues and solution were stirred in Erlenmeyer flasks for 20 min at room temperature. After stirring, the ear tissues were minced with experimental scissors and were rinsed with serum-free RPMI 1640 medium. The ear tissues were then incubated in 0.5 mg/mL collagenase type 1 (Sigma-Aldrich, St. Louis, MO, USA) in RPMI 1640 medium with slow rotation (130 rpm). After incubation, the solution containing ear cells was rinsed with cold PBS and passed through a 70 mm cell strainer. The filtered solution was centrifuged at 1300 rpm for 5 min. Cells were gathered and suspended in HBSS containing 10% FBS, 10% sodium azide, and 8% sodium bicarbonate. Infiltrated cells in ears were confirmed by flow cytometry. Surface molecules were stained with anti-CD4 (FITC), anti-CD8 (APC/Cy7), anti-CD11c (PE), anti-CD11b (APC), and anti-CD45R (PerCP/Cy5.5) (all from eBioscience, San Diego, CA, USA) for 30 min. Flow cytometry analysis was performed using BD Canto II instrument (BD Biosciences, San Diego, CA, USA).

### 2.8. Induction of the Anaphylactic Response by the Oral Administration of OVA

Mice were divided into naïve (*n* = 5), sham (*n* = 9), PCB (*n* = 9), and dexamethasone (*n* = 5) groups. To induce an allergic response, mice were sensitized with 20 *μ*g OVA in 2 mg/mL Imject Alum (Pierce, Rockford, IL) by intraperitoneal (i.p.) injection on day 0. Mice were then orally challenged with 50 mg OVA in saline every 3 days from day 17 for a total of six times. PCB extract (25 mg/kg) was administered orally daily from day 17 to 34 (Figure 1(b)). Diarrhea, anaphylaxis, and rectal temperature were measured as food allergy symptoms. To define diarrhea, the state of murine feces was observed and evaluated by visually monitoring once every 15 minutes for 1 hour after OVA challenge.

The criteria of diarrhea are the formlessness of stool and liquid shape (>80% water). The frequency, amount, and volume of stool were not used as an index showing a state of diarrhea. Temperature changes were measured with a rectal temperature probe (RET3) coupled to the Physitemp Thermalert Model TH-5 (Physitemp, Clifton, NJ). After OVA challenge, temperatures were measured every 15 minutes. Symptom scores were determined according to previously detailed criteria [30].

### 2.9. Measurement of Cytokine Levels Using ELISA

The mesenteric lymph node (mLN) from each mouse was removed aseptically. The mLNs were then homogenized into single-celled suspensions; 5 × 10^6^ cells/mL were added to RPMI medium, and cell viability was determined using trypan blue dye exclusion. Cells (200 *μ*L/well) were cultured in the presence or absence of OVA (100 *μ*g/mL) at 37°C for 72 h in a humidified incubator with 5% CO_2_ and 95% air. Cytokine assay kits (interferon *γ* [IFN-*γ*], IL-4, IL-5, IL-10, and IL-13, BD Pharmingen, San Diego, CA, and IL-17 and TGF-*β*, R&D Systems, Minneapolis, MN) were used to quantify cytokines following the manufacturer's instructions. The absorbance at 450 nm was measured using a microplate reader (Molecular Devices, Sunnyvale, CA).

### 2.10. Immunofluorescence Staining

To stain Foxp3 intracellularly, cells were washed in fluorescence-activated cell sorting (FACS) buffer (1% fetal calf serum and 0.1% NaN_3_ in PBS) and incubated with anti-CD16/32 antibody 2.4G2 for 5 min at 4°C to block Fc receptors (clone: 2.4G2) (BD Pharmingen, San Diego, CA). Surface CD4 molecules were stained with Phycoerythrin- (PE-) labeled anti-CD4 (clone: H129.19) (BioLegend, San Diego, CA) for 30 min at 4°C. The cells were then fixed and permeabilized using Foxp3 fixation/permeabilization concentrate and diluent (BD Biosciences, San Jose, CA) for 1 h at 4°C. After Fc receptors were blocked for 15 min, cells were stained using peridinin chlorophyll-a protein-Cy5.5- (PerCP-Cy5.5-) labeled anti-Foxp3 (clone: FJK-16s) (eBioscience, San Diego, CA) in FACS buffer at 4°C for 30 min. Data were acquired by flow cytometry using BD FACSCanto II (BD Biosciences) and analyzed using FlowJo software (Treestar, San Carlos, CA).

### 2.11. Quantitative Real-Time Reverse Transcription-Polymerase Chain Reaction (RT-PCR)

RNA from spleens and mLNs was extracted and purified using the RNeasy Mini Kit (Qiagen, Hilden, Germany), and cDNA was synthesized using the QuantiTect Reverse Transcription Kit (Qiagen). RT-PCR was performed in a Rotor-Gene Q 2plex System (Qiagen) as follows: 10 min at 95°C, followed by 45 cycles of 30 s at 95°C and 30 s at 62°C. The Assays-on-Demand kit (Qiagen) was used to analyze the expression of* HPRT* (housekeeping gene),* Foxp3*,* CTLA-4*,* AhR*, and* Granzyme B* (*GranB*). The sequences of the primers used are provided in Table 1.* HPRT* was used to normalize mRNA expression. The data are shown as relative delta delta CT (ΔΔCT); the fold-induction of each gene was calculated as follows: ΔThreshold cycle (ΔCt) = (Ct of target mRNA) − (Ct of HPRT); ΔΔCt = (ΔCt of mRNA in target gene) − (ΔCt of mRNA in control gene); fold-induction = 2^−ΔΔCt^.

### 2.12. Statistical Analysis

Data are presented as mean ± standard deviation (SD) of triplicate measurements. Differences between experimental data were analyzed using ANOVA followed by* F*-protected Fisher's least significant difference test, unpaired *t*-test, or one-way repeated measures of ANOVA followed by Tukey's or Dunnett's* post hoc* test. ND equals not detected. Statistical differences were presented as ^*∗*^ *P* < 0.05, ^*∗∗*^ *P* < 0.01, and ^*∗∗∗*^ *P* < 0.001.

## 3. Results

### 3.1. Effects of PCB Extract on the Symptoms of HDM-Induced AD

**Figure 2:**
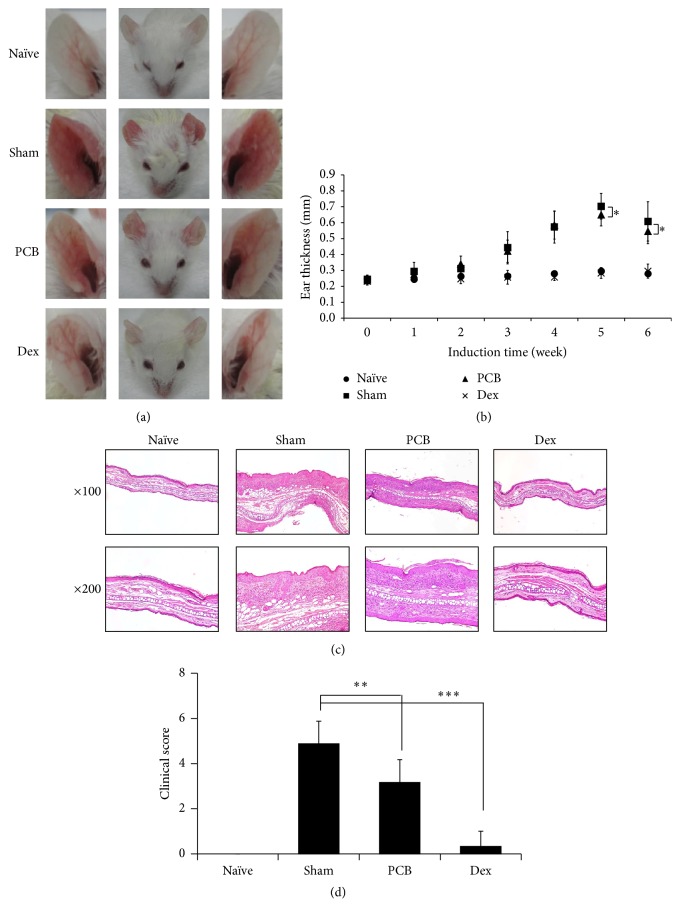
PCB extract alleviates the symptoms of DNCB/HDM-induced-atopic dermatitis. (a) Typical photographs of ear lesions developed by the treatments of DNCB and HDM on day 62. (b) Ear thickness was measured using digital thickness gauge on next day after HDM treatment from first challenge to sixth. (c) Ear lesions isolated from mice were fixed with 10% formalin solution and embedded in paraffin. To evaluate epidermal thickness and infiltrated inflammatory cells, H&E staining was performed and observed using a microscope at 100x and 200x magnification. (d) Clinical score was determined by criteria such as erythema/edema, scaling/dryness, and excoriation/hemorrhage to evaluate degree of severity in each ear lesion on day 62. Data are presented as the mean ± standard deviation (SD) of triplicate determinations and were analyzed using unpaired *t*-test. ^*∗*^ *P* < 0.05, ^*∗∗*^ *P* < 0.01, and ^*∗∗∗*^ *P* < 0.001 versus sham group.

**Figure 3:**
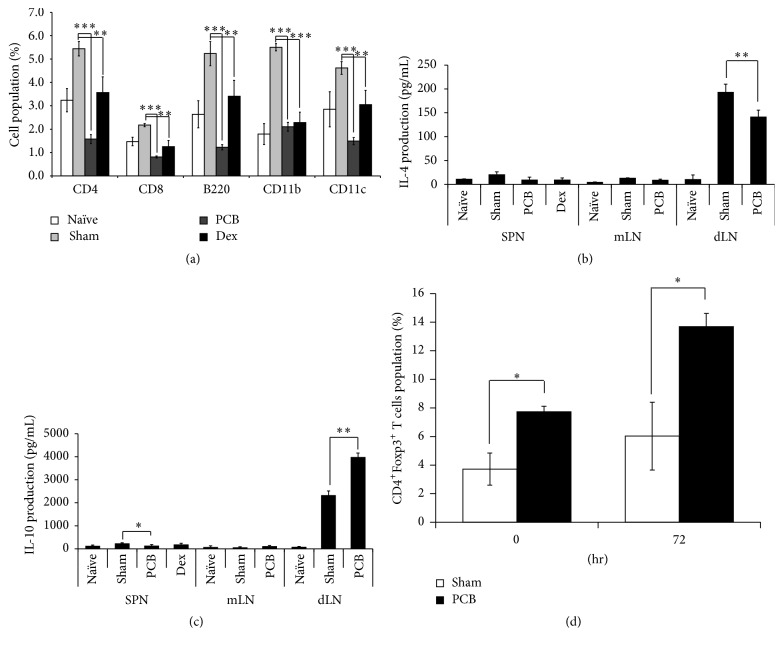
PCB extract suppressed infiltrated immune cell and Th2-related cytokine via Foxp3 regulation in a mouse model of AD. (a) For analyzed immune cells in ear, ear tissues were isolated single cells. The cells were stained with with anti-CD4 (FITC), anti-CD8 (APC/Cy7), anti-CD11c (PE), anti-CD11b (APC), and anti CD45R (PerCP/Cy5.5). These surface molecules of immune cells were measured by flow cytometry. To measure cytokines, the cells isolated from dLNs were restimulated with 100 *μ*g/mL HDM extract and cultured for 72 h. IL-4 (b) and IL-10 (c) were detected by ELISA. (d) To evaluate population of CD4^+^ Foxp3^+^ T cells, the cells isolated from dLNs were cultured with PMA (50 ng/mL) and Ionomycin (250 ng/mL). After staining with CD4 and Foxp3, the population of CD4^+^ Foxp3^+^ T cells was analyzed by flow cytometry at 0 h and 72 h. Each value is shown as mean ± SD (*n* = 3). ^*∗*^ *P* < 0.05, ^*∗∗*^ *P* < 0.01, and ^*∗∗∗*^ *P* < 0.001 compared with sham group. Data were analyzed using ANOVA followed by* F*-protected Fisher's least significant difference test.

In this study, we investigated the antiallergic effects of PCB extract in a mouse model of AD. The mice were orally administered with PCB extract at a dose of 25 mg/kg daily for 9 weeks. Ear thickness was evaluated on the day after the HDM challenge every week for 6 weeks. We found that PCB extract ameliorated ear thickness and epidermis thickness in models of HDM-induced AD (Figures 2(a)–2(c)). Furthermore, the clinical scores for criteria including erythema/edema, scaling/dryness, and excoriation/hemorrhage significantly reduced to 3.2 ± 1.0 points in the PCB extract group compared to 4.9 ± 1.0 points in the sham group (Figure 2(d)). We also examined the immune cells that infiltrated the ear in the AD models. The results indicated that, in ears of animals of the sham group, HDM treatment induced the infiltration of various immune cells such as CD4^+^ (T-helper cells), CD8^+^ (cytotoxic T cells), B220^+^ (B cells), CD11b^+^ (macrophages), and CD11c^+^ (dendritic cells) cells. However, the administration of PCB extract significantly reduced the infiltration of all these immune cells in the ears of animals in the PCB group (Figure 3(a)).

We next investigated how the PCB extract affected the Th2-related immune response in lymph nodes such as the spleen, mesenteric lymph node (mLN), and draining lymph node (dLN). The HDM-induced Th2-mediated immune responses were suppressed by the administration of PCB extract in each lymph node. In particular, HDM challenge strongly induced IL-4 production in the dLN, which was decreased by treatment with the PCB extract (Figure 3(b)). Meanwhile, administration of PCB extract enhanced the production of IL-10, which is a known anti-inflammatory cytokine in dLN. Since the PCB extract suppressed IL-4 production, enhanced IL-10 production in the lymph nodes, and decreased the population of immune cells in the inflammatory region, we investigated the induction of Tregs by analyzing the population of CD4^+^Foxp3^+^ T cells. We found that the administration of PCB extract promoted the expression of Foxp3 on CD4^+^ T cells in the dLN isolated from AD models (Figure 3(d)). These results demonstrated that the administration of PCB extract was able to attenuate the symptoms of AD by inducing Tregs in a mouse model of AD.

### 3.2. Effects of PCB Extract on the Response to OVA-Induced Food Allergies

**Figure 4:**
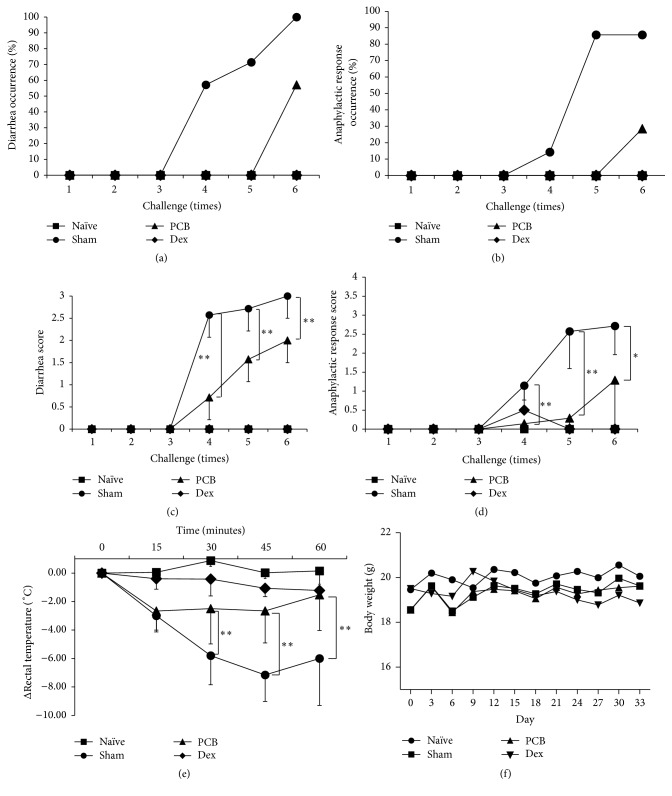
Effect of PCB extract on ovalbumin-induced diarrhea, anaphylactic response, and rectal temperature. OVA-induced food allergic symptoms were evaluated and scored by criteria for diarrhea and the anaphylactic response for 1 h after challenge with OVA. The diarrhea score (a), diarrhea occurrence (%, score ≥2) (c), anaphylactic response score (b), and anaphylactic response occurrence (%, score ≥2) (d) were evaluated on all challenges. (e) Rectal temperature was measured every 15 min for 1 h after the sixth OVA challenge. (f) Body weight during experiments. Data are presented as mean ± SD. Bars represent significant differences from the sham group at ^*∗*^ *P* < 0.05 and ^*∗∗*^ *P* < 0.01 versus sham. Data were analyzed using one-way repeated measures of ANOVA followed by Dunnett's* post hoc* test.

In this study, we investigated antiallergic effects of PCB extract in a mouse model of food allergy. We treated mice with PCB extract (25 mg/kg) on a daily basis by oral gavage. Food allergy symptoms were induced by oral challenges with OVA, and the following symptoms were evaluated. Any of the groups did not reach death by anaphylactic shock. Severe food allergy symptoms were observed in the sham group (scores: diarrhea, 3; anaphylactic response, 2.7) after the sixth oral challenge on day 34. On the contrary, OVA-induced diarrhea symptoms and the anaphylactic response were noticeably suppressed to 2 points and 1.3 in the PCB-treated group, respectively (Figures 4(a)–4(d)). Moreover, rectal temperature in the sham group decreased by −7.15°C compared with naïve mice. However, PCB extract treatment significantly ameliorated the OVA-induced decrease in rectal temperature to −2.65°C compared with control (Figure 4(e)). Although Dex completely blocked the food allergy symptoms, these mice group showed significant side effects of glucocorticoid, including serious size loss of splenocyte and mLN, and reduction of body weight by more than 7%. In contrast, PCB-treated group showed increase of body weights by more than 7% and no significant side effects (Figure 4(f)). This suggests that the oral administration of PCB extract attenuated food allergies by inhibiting diarrhea and the anaphylactic response without side effects.

**Figure 5:**
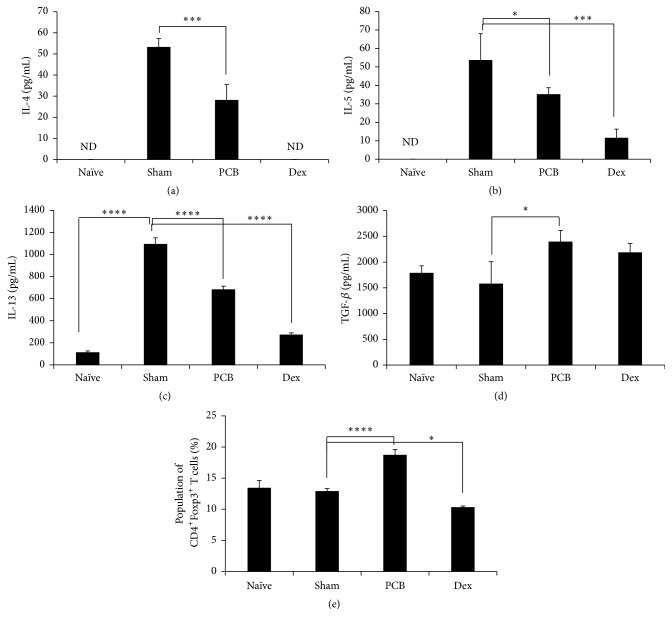
Immunomodulatory effects of PCB extract on cytokine expression patterns in mesenteric lymph nodes of food allergic mouse. Mice were sacrificed by cervical dislocation on day 35, and mLNs were isolated. mLNs were cultured in RPMI medium containing 10% fetal bovine serum for 72 h. Cytokines secreted from mLNs were quantified by ELISA. Interleukin- (IL-) 4 (a), IL-5 (b), IL-13 (c), and TGF-*β* (d) were measured. (e) The expression of Foxp3 in gated CD4^+^ cells was analyzed using flow cytometry. Data are presented as the mean of the percentage of Foxp3^+^CD4^+^ T cells from three independent experiments and as mean ± SD. Data were analyzed using one-way repeated measure ANOVA followed by Dunnett's* post hoc* test. Bars represent significant differences from the sham group at ^*∗*^ *P* < 0.05, ^*∗∗∗*^ *P* < 0.001, and ^*∗∗∗∗*^ *P* < 0.0001.

To prove the mechanism of how PCB controls food allergies, we next assessed cytokine expression in mLNs isolated from mice with OVA-induced food allergies. The Th2-related cytokines IL-4, IL-5, and IL-13 were increased in mLNs from food allergy-induced mice. However, this increase was inhibited significantly by treatment with PCB extract (Figures 5(a)–5(c)). In contrast, PCB treatment increased significantly in the TGF-*β* production which modulates the differentiation of T cells to Tregs (Figure 5(d)). Moreover, the PCB extract significantly increased the Foxp3^+^CD4^+^ Treg population in comparison with sham group (Figure 5(e)). This suggests that PCB extract regulates the abnormal Th2 dominance after allergic responses to food by increasing Tregs.

### 3.3. PCB Extracts Induced the Generation and Maintenance of Functional Foxp3^+^CD4^+^ Tregs

**Figure 6:**
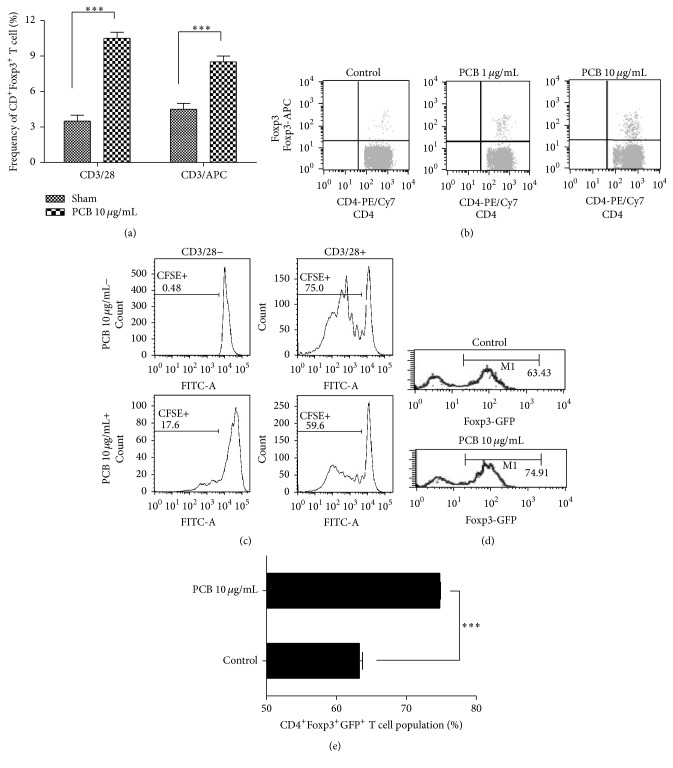
PCB extract affects the generation and functional Foxp3^+^ T regulatory cells and stabilizes the Foxp3 expression of regulatory T cells. (a) and (b) CD4^+^CD62L^+^ naïve T cell from splenocytes and mLN cells from BALB/c mice were cultured with 10 *μ*g/mL plate-bound anti-CD3 monoclonal antibody and 2 *μ*g/mL soluble anti-CD28 mAb, or with 10 *μ*g/mL plate-bound anti-CD3 mAb and APCs, in the presence of 0–10 *μ*g/mL PCB. After three days, the expression of Foxp3 in gated CD4^+^ cells was analyzed using flow cytometry. A plot from one representative experiment shows the frequency of Foxp3^+^CD4^+^T cells. (c) CD4^+^ T cells from each group were cocultured with CD4^+^CD62L^+^ naïve T cells labeled with CFSE (5 *μ*M), 10 *μ*g/mL plate-bound anti-CD3 mAb, and 2 *μ*g/mL soluble anti-CD28 mAb. The CFSE^+^ population was then analyzed using FACS. (d) Ag-specific GFP^+^ nTregs were sorted from the OT-II transgenic Foxp3-GFP knock-in mice and transferred to normal recipients. Five days after immunization, the transferred CD45.2^+^ cells were analyzed for Foxp3 expression using flow cytometry. (e) The numbers in M1 represent the percentage of Foxp3^+^ cells. The plot presents the mean percentage of CD45.2^+^Foxp3^+^ T cells of three independent experiments. Data are presented as the mean ± SD of triplicate determinations and were analyzed using unpaired *t*-test. ^*∗∗∗*^ *P* < 0.001 versus control.

To determine how PCB extract increased Foxp3^+^CD4^+^ Tregs specifically, CD4^+^CD62L^+^ naïve T cells were cotreated with TCR stimulation using anti-CD3/-CD28 mAbs or anti-CD3/APCs in the presence of PCB. The Foxp3^+^CD4^+^ T cell population in mLNs and splenocytes was then measured using FACS. Compared with control, PCB extract significantly increased the Foxp3^+^CD4^+^ Treg population with or without APC (Figure 6(a)). The PCB extract significantly increased the Foxp3^+^CD4^+^ Treg population in a dose-dependent manner (Figure 6(b)). We next examined whether PCB-induced Foxp3^+^CD4^+^ Tregs could suppress naïve CD4^+^ T cell proliferation. CFSE-labeled CD4^+^CD62L^+^ naïve T cells were cocultured with T cells, which had been precultured with PCB extract for 3 days, in the presence or absence of TCR stimulation for 3 days. CD4^+^ T cell proliferation was then analyzed by measuring the CFSE signal using FACS. CD4^+^CD62L^+^ T cells cocultured with PCB-treated CD4^+^ cells showed significantly reduced proliferation compared with control (59.6 versus 75%), presumably because of their increased Foxp3^+^ Treg population (Figure 6(c)).

To assess the effect of PCB extract on the maintenance of* Foxp3 *expression in Tregs, we isolated Foxp3-GFP by sorting CD45.2^+^ nTregs from the thymus of C57BL/6 mice. The stability of Foxp3^+^ Tregs in control group decreased significantly by 63.43% from 99% after 3 days. In contrast, the expression of Foxp3 in PCB-treated Tregs remained 74.91% (Figures 6(d) and 6(e)). These results indicated that PCB possesses activity that might directly generate and stabilize functional Foxp3^+^CD4^+^ Tregs.

### 3.4. PCB Extract Suppressed Cytokine Production in Effector T Cells

**Figure 7:**
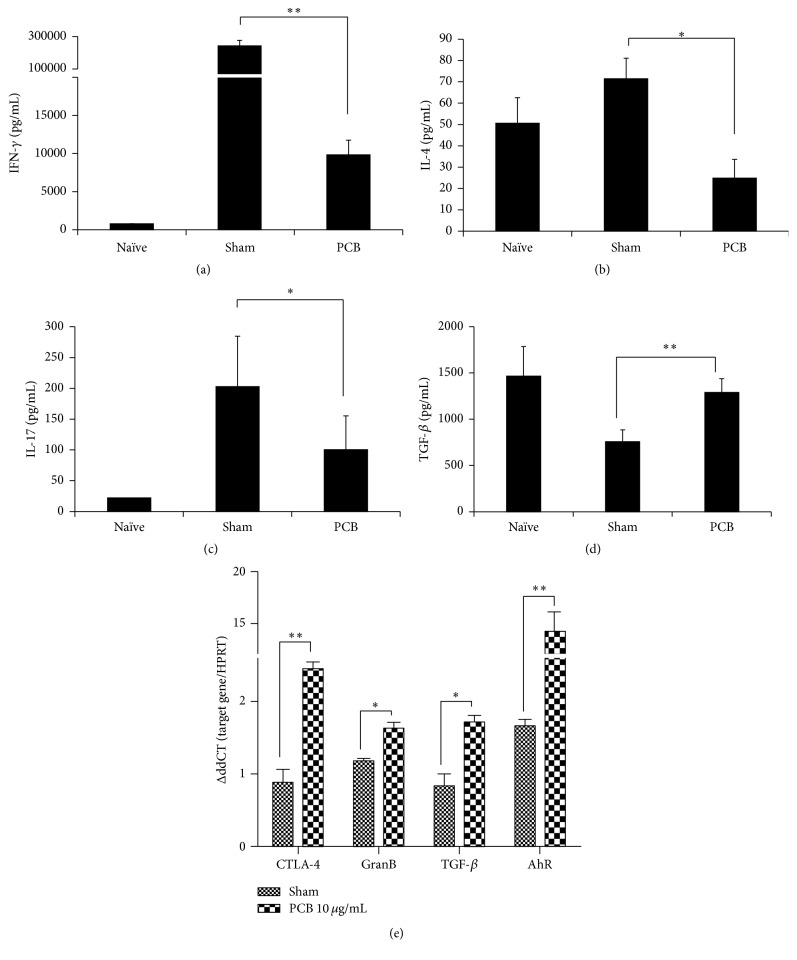
PCB extract contributed to an expanded population of inducible T regulatory cells. CD4^+^CD62L^+^ naïve T cells from splenocytes and mLN cells from BALB/c mice were cultured with 10 *μ*g/mL plate-bound anti-CD3 mAb and 2 *μ*g/mL soluble anti-CD28 mAb in the presence of 0–10 *μ*g/mL PCB. After three days, the levels of IFN-*γ* (a), IL-4 (b), IL-17 (c), and TGF-*β* (d) in the culture supernatants were measured using ELISA. Data are presented as the mean ± SD of triplicate determinations and were analyzed using ANOVA followed by* F*-protected Fisher's least significant difference test. ^*∗*^ *P* < 0.05; ^*∗∗*^ *P* < 0.01 versus OVA control. (e) The expression of Treg-associated molecules was compared between the sham and experimental groups. GranB: Granzyme B. Data are presented as the mean ± standard deviation (SD) of triplicate determinations and were analyzed using unpaired *t*-test. ^*∗*^ *P* < 0.05 and ^*∗∗*^ *P* < 0.01 versus OVA control.

We investigated whether the increased expression of CD4^+^Foxp3^+^ Tregs affected effector T cell function. CD4^+^CD62L^+^ T cells were isolated from the spleen and mLNs of BALB/c mice and were treated with anti-CD3 and anti-CD28 antibodies for 72 h. The levels of Th1 (IFN-*γ*), Th2 (IL-4), Th17 (IL-17), and Treg (TGF-*β*) cytokines in culture supernatants were then measured using ELISA. IFN-*γ*, IL-4, and IL-17 levels decreased in the PCB-treated compared with the TCR stimulated group, but TGF-*β* levels were increased in PCB-treated cells (Figures 7(a)–7(d)).

To further confirm the effect of PCB extract on Treg-related gene expression, CD4^+^CD62L^+^ T cells isolated from mouse spleens and mLNs were treated with anti-CD3 and anti-CD28 antibodies for 24 hr. The mRNA expression of Treg-associated genes was then measured (Figure 7(e)). PCB-treated inducible Foxp3^+^CD4^+^ T cells upregulated* CTLA-4*,* GranB*, and* TGF-β* expression compared with control. The expression of* AhR* also increased dramatically after treatment with PCB extract. This suggests that PCB extract could exert immunosuppressive effects on effector T cells by inducing functional Foxp3^+^CD4^+^ Tregs.

### 3.5. PCB Extract Directly Affects Naïve CD4^+^ T Cells to Induce Tregs via AhR Activation

**Figure 8:**
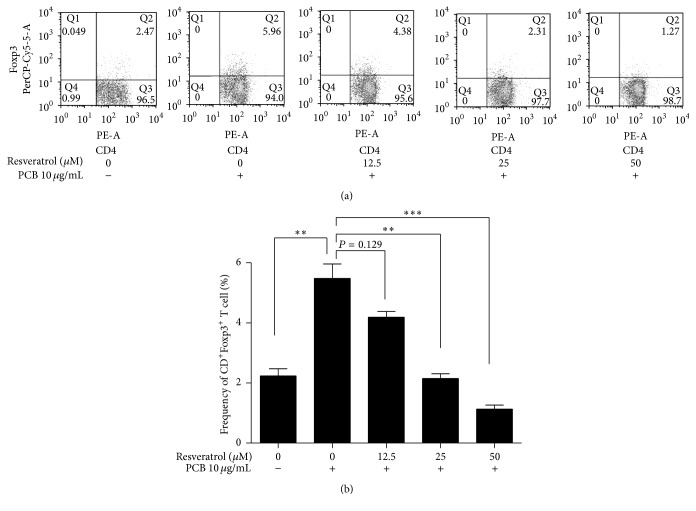
PCB extract induced the generation of inducible T regulatory cells via the aryl hydrocarbon receptor. (a) CD4^+^CD62L^+^ naïve T cell from splenocytes and mLN cells from BALB/c mice were stimulated with anti-CD3 and anti-CD28 antibodies in the presence of 10 *μ*g/mL PCB and the AhR antagonist resveratrol (12.5, 25, and 50 *μ*M) for 72 h. Foxp3^+^CD4^+^ T cell populations were then quantified using flow cytometry. (b) The plots show the mean population of Foxp3^+^CD4^+^ T cells from three independent experiments. Data are presented as the mean ± standard deviation (SD) of triplicate determinations and were analyzed using one-way repeated measures of ANOVA followed by Dunnett's* post hoc* test. ^*∗∗*^ *P* < 0.01 and ^*∗∗∗*^ *P* < 0.001 versus control.

AhR activation suppresses Th2-type cells and antibody responses and induces Foxp3^+^ Tregs [16, 31]. We measured whether the PCB-induced expression of* Foxp3* in naïve CD4^+^ T cells required AhR activation. Naïve CD4^+^CD62L^+^ T cells were cultured with resveratrol, an AhR antagonist [32], TCR stimulation, and PCB extract. Resveratrol decreased the PCB-induced Foxp3^+^CD4^+^ Treg population in a dose-dependent manner (Figure 8). This suggests that the induction of functional Foxp3^+^CD4^+^ Tregs by PCB extract required AhR activation.

## 4. Discussion

In this study, we focused on the antiallergic role of Tregs, which were induced by PCB extract, and whether these Tregs were associated with immune tolerance in AD and FA. This study demonstrated that the oral administration of PCB extract suppressed allergic symptoms without side effects and induced Foxp3^+^CD4^+^ Tregs. Furthermore, our data showed that PCB extract promoted the generation and maintenance of functional Foxp3^+^CD4^+^ Tregs in an AhR-dependent manner.

We confirmed that PCB extract effectively suppressed IL-4 production, which is the main Th2-mediated cytokine, and is a potent oral immune regulator of in HDM-induced experimental AD. We suggest that the underlying mechanism is induction of Tregs because the oral administration of PCB extract induced both CD4^+^Foxp3^+^ T cells and IL-10 production. IL-10-producing CD4^+^ T cells are well known as type 1 T regulatory (Tr1) cells, and an increase in their population could ameliorate AD [33, 34]. However, it was known that other immune cells such as macrophages, B cells, and dendritic cells could also promote IL-10 production with Treg or Tr1. IL-10-producing cells called type 2 macrophages, Breg, and tolerogenic DC also could suppress Th2-related immune responses in allergic disorders such as AD [35–37]. The main population of IL-10-producing cells in HDM-induced AD may be revealed through further studies.

Increase in Foxp3^+^CD4^+^ Tregs has been linked to inhibition of FAs initiated by Th2-related cytokines such as IL-4, IL-5, and IL-13 [38–40]. In this study, PCB extract administration significantly promoted oral tolerance via the generation of Foxp3^+^ Tregs and suppressed OVA-induced Th2-dominant immune responses in FA. Previous studies have demonstrated that the induction of Tregs could alleviate symptoms of food allergy, despite the relatively small proportion in immune cells [41]. Tregs inhibit the uncontrolled activation and expansion of T cells [15]. They directly suppress the activation and expansion of allergen-specific Th2 cells during allergic reactions [15, 42]. Many studies have reported the role played by TGF-*β* in mechanisms regulating the induction of tolerance at the gastrointestinal tract [43, 44]. TGF-*β* can efficiently convert naïve T cells into Tregs. TGF-*β* promote the nuclear translocalization of phosphorylated Smad3 which contributes to* Foxp3* initiation [45], a Treg-specific transcription factor. Our data also showed that oral administration of PCB extract increased TGF-*β* production and Foxp3 expression in mLN of the OVA-induced pathogenic mice. Moreover, PCB extracts stabilized the expression of inducible* Foxp3*. We therefore suppose that oral tolerance by administration of PCB may improve allergic disorders such as FA via the stable induction of TGF-*β*-dominated Foxp3 expression.

AhR plays an important role in T cell differentiation. Curcumin, a dietary AhR compound found in turmeric, attenuates inflammation in asthma and systemic lupus erythematosus by regulating the induction of Tregs [19, 46, 47]. In addition, I3C and indirubin increase the number of CD4^+^CD25^+^ Tregs and suppress chronic inflammatory diseases and/or autoimmunity* in vivo* [20]. In this study, PCB extract significantly increased AhR expression. Moreover, no increase in Foxp3 expression was found in the presence of the AhR antagonist resveratrol. This suggests that the immunosuppressive effects of PCB extract are mediated via AhR and that AhR expression and activation are essential for PCB-mediated Foxp3^+^ Treg generation. Some chemical constituents of PCB have been reported to be AhR agonists. For example, ergosterol regulated human CYP enzymes via AhR activation [48, 49] and hydrophobic triterpenes have a high affinity for AhR and induce its activation [26, 27, 50]. In this study, we found that CD4^+^Foxp3^+^ Tregs were efficiently generated by the hexane fraction of PCB containing ergosterol and triterpenes (*data not shown*). Consequently, it could be speculated that the ergosterol and triterpenes of PCB can induce Tregs. However, there remains a need to explore the putative compound(s) responsible for* Foxp3* expression in naïve CD4^+^CD62L^+^ T cells in the hexane fraction of PCB.

In conclusion, PCB extracts exerted potent immunosuppressive effects by potentiating the generation and maintenance of functional Foxp3^+^CD4^+^ Tregs in an AhR-dependent manner. Moreover, PCB-induced Foxp3^+^CD4^+^ Tregs effectively suppressed the symptoms of AD and FAs. Therefore, PCB extracts have potential as orally active immune suppressors for the treatment of hypersensitive immune responses disorders such as AD and FA.

## Figures and Tables

**Table 1:** Primer sequences used in this study.

Gene	Primer	Sequence
Hypoxanthine-guanine phosphoribosyl transferase	Sense	5′-CTGGTGAAAAGGACCTCTCG-3′
	Antisense	5′-TGAAGTACTCATTATAGTCAAGGGCA-3′

Forkhead box P3	Sense	5′-CAGCTGCCTACAGTGCCCCTAG-3′
	Antisense	5′-CATTTGCCAGCAGTGGGTAG-3′

Cytotoxic T-lymphocyte antigen 4	Sense	5′-AGA ACC ATG CCC GGA TTC TG-3′
	Antisense	5′-CAT CTT GCT CAA AGA AAC AGC AG-3′

Granzyme B	Sense	5′-CTC CTA AAG CTG AAG AGT AAG G-3′
	Antisense	5′-TTT AAA GTA GGA CTC ACA CTC CC-3′

Aryl hydrocarbon receptor	Sense	5′-TGCACAAGGAGTGGACGA-3′
	Antisense	5′-AGGAAGCTGGTCTGGGGTAT-3′

## Abbreviations

AD: Atopic dermatitis
APCs: Antigen-presenting cells
Dex: Dexamethasone
DfE: * Dermatophagoides farinae* extracts
dLN: Draining lymph node
DNCB: Dinitrochlorobenzene
Foxp3: Forkhead box P3
H&E: Hematoxylin and eosin
IFN: Interferon
Ig: Immunoglobulin
IL: Interleukin
iTreg: Inducible Treg
mLN: Mesenteric lymph node
OVA: Ovalbumin
PCB: * Poria cocos*
ROR*γ*T: Retinoic acid-related orphan receptor *γ*T
TCR: T cell receptor
Teff cells: Effector T cells
TGF-*β*: Transforming growth factor-*β*
Th: T-helper
Th17 cells: IL-17-producing Th cells
Tregs: Regulatory T cells.
